# Instrumental aversion coding in the basolateral amygdala and its reversion by a benzodiazepine

**DOI:** 10.1038/s41386-021-01176-2

**Published:** 2021-09-07

**Authors:** Philip Jean-Richard-dit-Bressel, Jenny Tran, Angelos Didachos, Gavan P. McNally

**Affiliations:** School of Psychology, UNSW Sydney, Kensington, NSW Australia

**Keywords:** Operant learning, Motivation

## Abstract

Punishment involves learning the relationship between actions and their adverse consequences. Both the acquisition and expression of punishment learning depend on the basolateral amygdala (BLA), but how BLA supports punishment remains poorly understood. To address this, we measured calcium (Ca^2+^) transients in BLA principal neurons during punishment. Male rats were trained to press two individually presented levers for food; when one of these levers also yielded aversive footshock, responding on this punished lever decreased relative to the other, unpunished lever. In rats with the Ca^2+^ indicator GCaMP6f targeted to BLA principal neurons, we observed excitatory activity transients to the footshock punisher and inhibitory transients to lever-presses earning a reward. Critically, as rats learned punishment, activity around the punished response transformed from inhibitory to excitatory and similarity analyses showed that these punished lever-press transients resembled BLA transients to the punisher itself. Systemically administered benzodiazepine (midazolam) selectively alleviated punishment. Moreover, the degree to which midazolam alleviated punishment was associated with how much punished response-related BLA transients reverted to their pre-punishment state. Together, these findings show that punishment learning is supported by aversion-coding of instrumental responses in the BLA and that the anti-punishment effects of benzodiazepines are associated with a reversion of this aversion coding.

## Introduction

Our values and behaviors are shaped by our experiences. Organisms, from invertebrates to mammals, possess two highly-conserved learning systems to dynamically adapt behavior to aversive conditions: Pavlovian fear learning and instrumental aversive learning. Pavlovian fear learning allows organisms to learn predictive relationships between environmental stimuli and aversive outcomes (Stimulus–Outcome [S-O] or conditioned stimulus – unconditioned stimulus [CS-US] associations), endowing those stimuli with the ability to elicit involuntary and highly stereotyped defensive reflexes such as freezing [1, 2]. By contrast, when the behavior causes aversive outcomes (punishment), we can learn the instrumental association between actions and their aversive consequences (Action–Outcome associations) to voluntarily and flexibly withhold these actions to avoid detriment [3]. Although behavioral and neural manipulation studies show that these two forms of aversive learning are distinct [3–6], both depend on the basolateral amygdala (BLA).

BLA has long been ascribed a critical role in Pavlovian fear learning [7]. Single unit recording and calcium imaging studies show that BLA principal neurons are activated by response-independent aversive outcomes (USs) [8, 9]. This activation instructs synaptic plasticity as a substrate for Pavlovian CS-US association formation and CS salience augmentation [9, 10]. Notably, across Pavlovian conditioning, there is a remapping of CS-evoked activity in ensembles of BLA principal neurons so that CS-evoked activity increasingly resembles US-evoked activity, with these changes in CS activity predicting the extent of CS-elicited fear reflexes [8].

The BLA also contributes to punishment learning. Reversible inactivation or lesion of BLA impairs punishment learning and the expression of punishment avoidance independently of its role in Pavlovian conditioned fear [6, 11, 12]. Yet how BLA activity during punishment supports punishment learning is poorly understood. This is because most existing studies of instrumental aversion have focused on active avoidance of fear CSs [13] and have not isolated instrumental Action–Outcome contingencies that underpin instrumental punishment learning. So, whether punishment, like fear, is accompanied by alterations in BLA activity around punishers and their causal antecedents (i.e., punished responses) during punishment learning remains unknown.

Here we addressed these issues. We examined BLA principal neuron activity during punishment and assessed how this activity might support punishment avoidance. To do this, we used fiber photometry to measure BLA activity across a task that selectively promotes punishment learning. We also examined how anxiolytic benzodiazepines affected BLA activity during punishment. Benzodiazepines have robust, well-established anti-punishment effects via their actions as positive allosteric modulators of GABA_A_ receptors. Systemic and intra-BLA administration of benzodiazepines selectively increases punished behavior [14–16]. BLA activity during punishment sessions was measured following systemic administration of benzodiazepine midazolam.

## Materials and methods

### Subjects

Subjects were experimentally naive male Long Evans rats (327–610 g) aged 9–13 weeks, obtained from the colony maintained by UNSW Sydney, Australia. Animals were housed in groups of four in plastic cages in a climate-controlled colony room maintained on a 12 h light–dark cycle. They were given 10–15 g food/day with free access to water to maintain weight at ~90% of their free-feeding weight. Procedures were approved by the Animal Care and Ethics Committee at UNSW and conducted in accordance with the Australian Code for the Use and Care of Animals (NHMRC, 2013). Male rats were used as females tend to exhibit greater, generalized response suppression attributable to fear and not punishment learning [4, 17].

### Apparatus

See Supplemental Material for full description. Briefly, eight identical chambers (24 [length] × 30 [width] × 21 [height] cm; MedAssociates, St Albans, VT, USA) were located inside individual light and sound-attenuating cabinets (40 × 56 × 56 cm). AAV5-CaMKIIα-GCaMP6f-WPRE-SV40 (1.23 ×1013 vp/ml) (Penn Vector Core, Philadelphia, PA, USA) was used to target principal neurons [18]. Fiber Photometry recordings were performed using Fiber Photometry Systems from Doric Lenses and Tucker Davis Technologies (TDT, Alachua, FL, USA).

### Procedure

#### Experiment 1: Behavioral characterization of punishment

##### Lever-press Training

There were two sessions of FR1 lever-press training; both levers (left, right) were extended and pressing was reinforced with pellet delivery on an FR1 schedule. A lever retracted after 25 presses on it. The session ended after 1 h had elapsed or if both levers had retracted. Rats that failed to acquire lever-pressing were manually shaped in the 2nd FR1 session.

Rats then received seven daily, 40 min lever-press training sessions. Levers were presented individually for 5 min in alternating fashion; one lever was extended while the other was retracted (4 trials per lever across 40 mins). Lever-pressing was reinforced with pellets on a VI30s schedule. First lever to be extended (left, right) was fully randomized.

##### Punishment

Rats received six daily, 40 min punishment sessions. These were identical to lever-press training, except that responses on one lever (punished lever) delivered a 0.5 s, 0.4 mA footshock on an FR10 schedule (independent of pellet outcome). If a response was due to deliver both pellet and shock (coincidental), both were delivered. Each animal was assigned the same punished lever throughout punishment (left or right, counterbalanced across animals). The other lever remained unpunished.

##### Choice Test

Rats were then given a 10 min choice test where both levers were presented concurrently. No shocks were delivered and presses on either lever delivered pellets on a shared VI60s schedule, so there was no advantage to pressing either lever exclusively or a combination of both levers.

#### Experiment 2: BLA activity during punishment

Behavioral training commenced four weeks after surgery (Supplemental Material) to allow recovery and sufficient GCaMP expression. All analyzed subjects (*n* = 14) received lever-press training and six days of punishment, as per Experiment 1. Rats then received choice (identical to Experiment 1) and/or midazolam tests (described below); some rats received both choice and midazolam tests (*n* = 5), some only received choice test (*n* = 5), and some only received midazolam tests (*n* = 4). Thus, analyses (behavior, neural activity) for choice tests are *n* = 10, whereas analyses for midazolam tests are *n* = 9. Choice test was always conducted following punishment sessions and prior to midazolam tests, as applicable.

Subjects were connected to fiber optic patch cables and received light stimulation (405 nm, 465 nm) to allow BLA neural recordings for the last three days of lever-press training (two days for habituation, followed by one for lever-press training recording), punishment sessions, choice and midazolam tests.

##### Midazolam tests

0 mg (0.9% w/v saline), 0.3 mg or 1 mg/ml/kg of midazolam (MDZ; Hypnovel, Roche, diluted with 0/9% w/v saline) was injected i.p. fifteen minutes prior to punishment session. Rats received each dose across three tests (within-subject, order counterbalanced). Each MDZ test day was preceded by an injection-free punishment session. MDZ has well-documented anti-punishment effects at low doses, but sedative effects at higher doses [14, 19]. Doses for the current study match those used by Killcross et al. [16] who found anti-punishment effects of MDZ at 0.3 mg/kg i.p. and sedative effects at 1 mg/kg. However, other studies have observed anti-punishment effects at doses as high as 1.5 mg/kg [20], providing some basis for expecting anti-punishment effects across doses used here.

### Data Analysis

Behavior was analyzed using within-subject planned contrasts in PSY statistical software [21]. All fiber photometry data were analyzed using custom MATLAB scripts. The Type I error rate (α) for all analyses was controlled at 0.05. Only rats with correct AAV expression and fiber placement in BLA were included (Supplemental Material).

#### Behavior – lever-pressing

The behavioral dependent variables were lever-press rates on punished and unpunished levers. Punishment and choice test lever-press rates were analyzed using orthogonal contrasts for lever (punished vs unpunished), session (linear, quadratic), and interaction contrasts. Lever-training data was analyzed separately from punishment session data. Latencies to first lever-press (averaged across trials per session) were analyzed using the same contrasts. Lever-press rates across MDZ test were analyzed using simple effect contrasts comparing an MDZ dose (0.3 mg or 1 mg) against control (0 mg).

Ratios of lever-pressing were used to assess self-normalized change in lever-pressing. An elevation ratio was used to compare lever-press (LP) rates for a lever under a dose of midazolam (0.3 mg or 1 mg MDZ) against control (0 mg, i.e., saline). These were calculated once per lever as follows:

$$Elevation\;ratio = \frac{{MDZ\;session\;LP\;rate}}{{MDZ + Saline\;session\;LP\;rates}}$$

Elevation ratios are bounded from 0 to 1. A score of 0.5 indicates no change in lever-press rate for a lever following MDZ relative to saline, a score higher than 0.5 indicates increased lever-pressing relative to saline, and a score lower than 0.5 indicates decreased lever-pressing relative to saline. Elevation ratios were compared against the null ratio of 0.5 using single mean t-tests.

A suppression ratio was used to compare punished lever-press (PunLP) rates across midazolam tests (0 mg, 0.3 mg, 1 mg) relative to pre-punishment training (T), calculated as follows:

$$Suppression\;ratio = \frac{{Test\;PunLP\;rate}}{{Test + T\;session\;PunLP\;rates}}$$

Suppression ratios are bounded from 0 to 1. A score of 0.5 indicates no difference in punished lever-press rate during an MDZ test session relative to training, a score higher than 0.5 indicates more lever-pressing relative to training, and a score lower than 0.5 indicates less lever-pressing relative to training.

#### Behavior – location and immobility

Video recordings from the last day of lever-press training and selected punishment sessions (P1, P4, P6) were imported into EthoVision XT 10 (Noldus, Wageningen, Netherlands) to define animal center-point. To quantify spatial position, zones (4 [length] × 6 [width] cm) around the punished and unpunished levers were defined as punished and unpunished zones. The cumulative time spent in each zone per 5 min trial was calculated in seconds. Freezing was measured as the proportion of 5 min trials spent immobile. One rat was excluded from spatial location and immobility analyses due to a recording malfunction. This resulted in a final group size of *N* = 7 for analyses of spatial location and immobility, and *N* = 8 for all other analyses.

#### Fiber photometry – signal processing

Ca^2+^-dependent (465nm-related) and isobestic (405nm-related) signals and event timestamps were extracted into MATLAB, and signals during logged disconnections were discarded. Each signal was low-pass (3 Hz) and band-stop filtered (1.9–2.2 Hz) to remove high-frequency noise identified via Fast Fourier Transform. The isobestic signal was linearly regressed onto the Ca^2+^-dependent signal to create a fitted isobestic signal, and a normalized fluorescence change score (dF/F) was calculated using the standard formula:

$$\frac{{\left( {{{{{{{{\mathrm{Ca}}}}}}}}^{2 + }{{{{{{{\mathrm{dependent}}}}}}}}\;{{{{{{{\mathrm{signal}}}}}}}}--{{{{{{{\mathrm{fitted}}}}}}}}\;{{{{{{{\mathrm{isobestic}}}}}}}}} \right)}}{{{{{{{{{\mathrm{fitted}}}}}}}}\;{{{{{{{\mathrm{isobestic}}}}}}}}}}$$

This motion-artifact-corrected dF/F was detrended via a 600 s moving average, such that each 10 min period had a mean dF/F of 0. All peri-event activity used in analyses were derived from this detrended dF/F.

#### Fiber photometry – event-related activity

The key dependent variable was BLA activity transients around aversive footshock, and punished and unpunished lever-presses. dF/F from −3s to +7 s around lever-presses alone (i.e., those not yielding footshock or pellet outcomes) and footshocks (necessarily concomitant with punished lever-press) were collated. Peri-event activity kernels were obtained by normalizing each trial waveform according to its sum square deviation from 0 [22]. Activity kernels were averaged per subject; all analyses used mean activity kernels per subject. Due to the scarcity of punished lever-presses and shock deliveries towards the end of punishment, sessions P5 and P6 were combined to obtain more accurate peri-event activity traces per subject.

To determine significant transients, 95% confidence intervals (CI) around activity kernels were derived via bootstrapping [23]. Bootstrapped means were obtained by randomly resampling from subject mean waveforms with replacement (1000 iterations). CI limits were derived from 2.5 and 97.5 percentiles of bootstrap distribution, expanded by a factor of $\sqrt {n/(n - 1)}$ [23]. A significant transient was identified as a period that CI limits did not contain 0 (moving average baseline) for at least 1/3secs (low-pass filter window; Jean-Richard-dit-Bressel et al., 2020).

Punished and unpunished lever-press transients within a session were directly compared against each other by bootstrapping the within-subject difference waveform (mean punished–mean unpunished waveform per subject). Punished lever-press transients across MDZ tests were directly compared against each other by bootstrapping the within-subject difference of test waveforms (e.g., mean 0mg–mean 0.3 mg waveform per subject).

Supplemental analyses of peri-event activity using a pre-event baseline were also conducted (Fig. S2). Trial waveforms were zeroed to average activity −5 to −3s before each event trial prior to normalization and bootstrap analysis. This approach was not adopted more generally as the continuous nature of the task meant a baseline period could include events that problematically affect baselining of that trial. This concern was avoided by using a moving average baseline (encompassing equal parts punished and unpunished trials), described above, for all non-supplementary analyses.

#### Fiber photometry – kernel similarity

Similarity between lever-press and shock activity kernels across sessions was quantified by deriving a normalized fit score per kernel comparison. Fit score was calculated as the dot product of two waveform vectors (each normalized according to sum square deviation from 0). This score can range from −1 to 1. A fit above 0 indicates the two waveforms deviate from baseline in a similar way across the comparison window (1 = identical waveforms), whereas a negative fit indicates the two waveforms deviate from baseline in opposite directions (−1 = mirror opposite waveform). As each kernel is normalized, this method is specifically sensitive to differences in the specific shape of waveforms across the event window, not waveform magnitude.

To determine whether lever-press and shock waveforms across sessions were significantly similar (fit > 0) or inverse (fit < 0) to each other, 95% CI limits for fits were obtained from 2.5 and 97.5 percentiles of bootstrapped fit distributions (fits of randomly resampled mean waveforms; 1000 iterations). Two activity kernels were identified as significantly similar if the fit CI was entirely above 0, or significantly inverse if fit CI was entirely below 0.

To visualize the overall similarity/dissimilarity of activity kernels across sessions, fit scores were converted into fit distances (1 – fit [perfect fit = 0, perfect inverse = 2]). Kernel coordinates in 2D space were obtained via multidimensional scaling (MATLAB *mdscale* function, criterion = metricstress), using fit distances as input. Stress was 0.038, indicating an excellent representation of kernel similarity/dissimilarity within 2D space.

#### Fiber photometry – relative similarity of punished lever activity

To assess the relationship between BLA lever-press activity and punishment avoidance across MDZ tests, a relative fit of MDZ test lever-press activity to late punishment vs. pre-punishment was calculated per subject. Specifically, relative fit was the normalized fit (as described above) of a subject’s MDZ test punished lever-press activity against their late punishment (P5-6) punished lever-press activity, minus the normalized fit of MDZ test activity against their pre-punishment (T) punished lever-press activity (P5-6 fit – T fit). This score captures the degree a subject’s MDZ punished lever-press kernel conforms to learned punishment vs. pre-punishment kernels. A positive relative fit score indicates an MDZ test kernel is closer to late punishment than pre-punishment, whereas a negative relative fit score indicates a kernel more similar to pre-punishment.

The relationship between relative fit and suppression ratio across MDZ tests was assessed using linear regression (GraphPad Prism 9, San Diego, CA, USA).

## Results

### Experiment 1: Behavioral characterization of punishment

Rats were initially trained to respond on two individually-presented levers (5 min alternating trials) for food, each lever reinforced on a VI30s schedule. This VI30 schedule remained in effect for the remainder of the experiment. The mean ± standard error of the mean (SEM) lever-press rates at the end of training (T) are shown in Fig. 2B (left panel). There was no difference between to-be punished and to-be unpunished lever-pressing (*F*_*(1,7*)_ = 0.614, *p* = 0.459) (Fig. 2B, left panel [T]; Fig. S1). There were also no differences in latencies to initially respond on to-be punished and to-be unpunished levers (*F*_(1,7)_ = 0.275, *p* = 0.616) (Fig. 2B, right panel [T]).

Next, during punishment sessions (P1 to P6), one of these responses was punished via pairings with a 0.4 mA footshock on an FR10 schedule [24]. Animals exhibited robust punishment avoidance, pressing the punished lever less than the unpunished lever (lever main effect: *F*_(1,7)_ = 77.079, *p* < 0.001). There was a lever × session interaction (linear: *F*_(1,7)_ = 34.803, *p* < 0.001, quadratic: *F*_(1,7)_ = 6.780, *p* = 0.035); punished lever-presses initially decreased followed by a modest increase across sessions (session quadratic [punished only]: *F*_(1,7)_ = 14.768, *p* = 0.006), whereas unpunished lever-presses increased across sessions (session linear [unpunished only]: *F*_(1,7)_ = 25.706, *p* < 0.001).

**Fig. 1:**
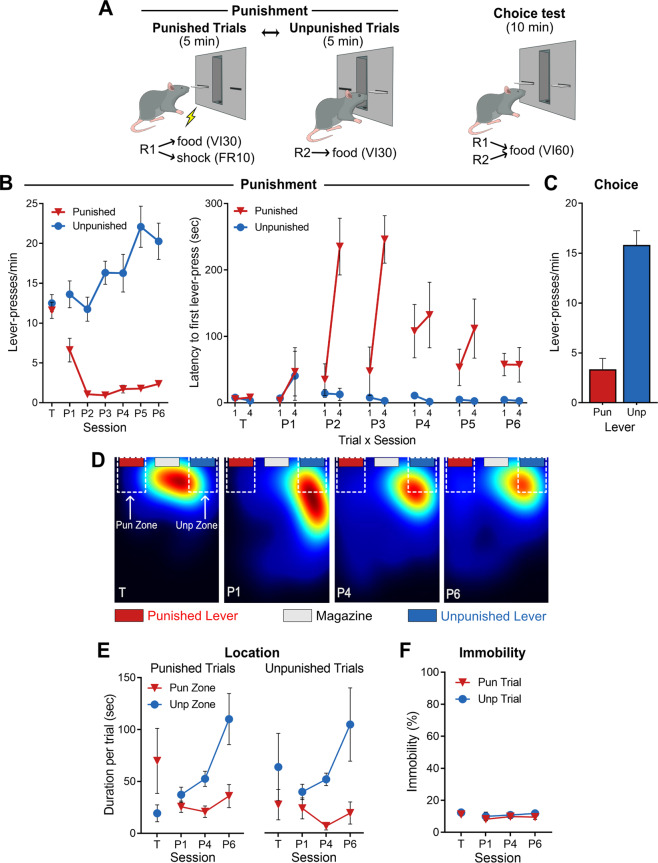
Punishment task and behavior. **A** Punishment task. *Left:* After training rats to press two individually-presented levers (5 min alternating trials) for food (30 s variable interval [VI30s] schedule), rats receive punishment sessions where presses on the punished lever also yielded footshock (FR10 schedule). *Right:* During choice test, both levers are presented to assess lever preference. **B** Left: Mean ± SEM lever-press rate for last session of lever-press training (T) and across punishment sessions (P1-P6) (*n* = 8). Prior to punishment, rats responded equally on both levers. Across punishment sessions, rats suppressed punished, but not unpunished, responding. Right: Mean ± SEM latency to first lever-press for first (1) and last (4) trial per session. Rats were slower to press the punished lever relative to unpunished lever across punished sessions, indicative of learned avoidance. **C** During choice test, rats preferred pressing the unpunished lever over the punished lever. **D** Example heatmaps of rat location during lever-press training (T) and punishment sessions (P1, P4, P6). Areas immediately around the punished and unpunished levers were designated as Pun Zone and Unp Zone, respectively. **E** Mean ± SEM time spent in punished and unpunished zones during punished and unpunished trials (left and right, respectively) across lever-press training (T) and punishment sessions (P1, P4, P6) (*n* = 7). Before punishment, rats were preferentially located in the extended lever’s zone. During punishment, rats tended to be in the unpunished zone, regardless of which lever was extended. **F** Mean ± SEM immobility across punished and unpunished trials. Low levels of immobility (proxy for freezing) were observed across sessions, suggesting low levels of Pavlovian fear in this task.

Punishment also affected latencies to first lever-press across sessions (Fig. 1B, right panel; Fig. S1). Rats were slower to respond on the punished lever than the unpunished lever (*F*_(1,7)_ = 36.307, *p* = 0.001). A lever × session interaction was also observed (quadratic: *F*_(1,7)_ = 17.015, *p* = 0.004); latencies to respond on the punished lever increased and then decreased across sessions (*F*_(1,7)_ = 20.570, *p* = 0.003), whereas latencies to respond on the unpunished lever did not significantly change across sessions (*F*_(1,7)_ = 0.819, *p* = 0.396).

Finally, rats received a choice test, during which both levers were extended but neither was punished. Animals made more unpunished than punished lever-presses (*F*_(1,7)_ = 70.212, *p* < 0.001) (Fig. 1C; Fig. S1), indicating a preference for unpunished over punished lever, despite the absence of shock on these tests.

We also assessed spatial location across punishment. Prior to punishment, there was no overall difference in time spent in punished versus unpunished zones (*F*_(1,6)_ = 0.044, *p* = 0.841) or time spent around levers during punished versus unpunished trials (*F*_(1,6)_ = 0.003, *p* = 0.958) (Fig. 1D-E). However, there was a significant interaction between zone and trial (*F*_(1,6)_ = 6.386, *p* = 0.045); rats tended to stay in the zone of whichever lever was extended, although follow-up simple effect analyses on zone per trial were insignificant (all *F*_(1,6)_ ≤ 5.689, *p* ≥ 0.054).

Punishment changed these spatial preferences (Fig. 1D, E). During punishment sessions, rats spent more time in unpunished compared to punished lever zones (*F*_(1,6)_ = 11.88, *p* = 0.014), and spent less time around levers during punished trials than unpunished trials (*F*_(1,6)_ = 8.875, *p* = 0.025). There was no trial x zone interaction (*F*_(1,6)_ = 0.234, *p* = 0.646); rats spent more time in unpunished zones during both punished (*F*_(1,6)_ = 7.208, *p* = 0.036) and unpunished (*F*_(1,6)_ = 7.703, *p* = 0.032) trials. The extent of this spatial preference for the unpunished over the punished zone increased across punishment sessions (*F*_(1,6)_ = 8.479, *p* = 0.027); rats increasingly spent more time around the unpunished lever (*F*_(1,6)_ = 8.753, *p* = 0.025), but did not significantly change time spent near the punished lever (*F*_(1,6)_ = 0.136, *p* = 0.725) across punishment sessions. This shows emergence of a preference for spending time near the unpunished lever, regardless of whether the punished or unpunished lever was extended. Visual observations suggested that animals spent more time exploring the chamber during punished trials, frequently returning to the unpunished zone to check for the unpunished lever.

#### Immobility

Pavlovian aversive learning instructs Stimulus-Outcome (CS-US) associations [2], which control innate behaviors such as freezing [1]. Punishment instructs Action–Outcome associations, controlling voluntary withholding of a specific action [25]. However, involuntary, reflexive behaviors like freezing may interfere with and suppress lever-pressing, thereby confounding the measure of punishment with fear [3]. We directly measured immobility across punishment sessions to determine whether and when immobility may have contributed to the suppression we observed. Immobility was low across all sessions (Fig. 1F). There were no differences in immobility during punished and unpunished trials prior to punishment (*F*_(1,6)_ = 1.852, *p* = 0.222) (Fig. 1F). During the punishment, there was no main effect of trial (*F*_(1,6)_ = 1.569, *p* = 0.257), and no trial × session interaction (*F*_(1,6)_ = 0.050, *p* = 0.830). This shows that immobility and freezing were not the cause of lever-pressing effects within this task [11].

### Experiment 2: Basolateral amygdala calcium transients across the punishment

**Fig. 2:**
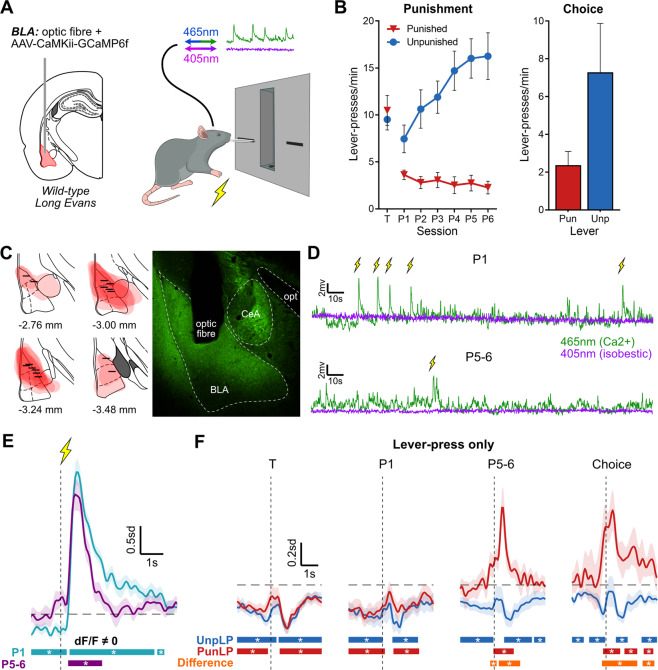
Basolateral amygdala activity during punishment. **A** Fiber photometry to assess basolateral amygdala (BLA) activity. Left: Rats received unilateral optic fiber implant and application of AAV-CaMKii-GCaMP6f into BLA. Right: BLA neuron activity was measured throughout punishment. **B** Behavior of BLA photometry animals. Left: Mean ± SEM lever-press rate on punished and unpunished levers for last day of lever-press training (T) and punishment sessions (P1–P6) (*n* = 14). *Right*: Mean ± SEM lever-press rate during choice test (*n* = 10). **C** Left: GCaMP6f expression and fiber tip location for animals included in analysis (*n* = 14). Right: Example BLA placement. **D** Example of 465nm- and 405nm-related signals during early (P1; top) and late (P5-6; bottom) punishment sessions, with shock deliveries times. **E** Mean ± SEM of subject-based (*n* = 14) BLA activity kernel around shock deliveries during early (P1; teal) and late (P5-6; purple) punishment. Bars at bottom of graph indicate significant deviations from baseline (dF/F ≠ 0), determined via bootstrapped confidence intervals (95% CI). Vertical dashed line indicates shock onset, horizontal dashed line indicates baseline (dF/F = 0). **F** Mean ± SEM BLA activity kernel around punished (PunLP; red) and unpunished (UnpLP; blue) lever-presses (no outcome) across training (T), punishment (P1, P5-6) and choice test. Bars at bottom of graph indicate significant deviations from baseline (95% CI) for PunLP and UnpLP, and significant differences between punished and unpunished kernels (Difference; orange). Vertical dashed lines indicate time of lever-press, horizontal dashed lines indicate baseline (dF/F = 0).

We examined population-level BLA principal neuron calcium (Ca^2+^) transients during punishment learning. An AAV encoding GCaMP6f under control of the CaMKIIα promoter was applied to the BLA to express the genetically-encoded Ca^2+^ sensor in BLA principal neurons [18]. GCaMP6f fluorescence was measured via an optic fiber cannula implanted in BLA (Fig. 2A, C).

The mean ± SEM lever-press rates for a final lever-press training session (T) and punishment sessions (P1–P6) are shown in Fig. 2B. There were no differences in lever-press rates for to-be punished and unpunished levers at end of lever-press training (*F*(1,13) = 0.466, *p* = 0.507). During punishment, animals pressed the unpunished lever more than punished lever (lever main effect: *F*(1,13) = 30.72, *p* < 0.001). This difference increased across sessions (lever × session interaction: *F*(1,13) = 22.56, *p* < 0.001); unpunished lever-pressing increased (*F*(1,13) = 36.31, *p* < 0.001) whereas punished lever-presses decreased (*F*(1,13) = 6.12, *p* = 0.028) across punishment. For animals that underwent choice test (*n* = 10; Fig. 2B [right]), there was a significant preference for unpunished over punished levers (*F*(1,9) = 18.75, *p* = 0.002).

The first question was how BLA activity related to delivery of punishment. We analyzed Ca^2+^ transients using 95% confidence intervals with a consecutive threshold of 0.33 s [23]. The BLA exhibited robust excitatory Ca^2+^ transients to response-generated footshock across punishment (Fig. 2E), showing recruitment of BLA principal neurons by the punisher. This is similar to previous reports of excitatory Ca^2+^ transients to response-independent footshocks in studies of Pavlovian fear [8, 9].

Punishment learning involves encoding the instrumental relationship between actions and their aversive outcomes. So, the next question was whether punishment changed BLA encoding of punished responses (lever-presses). Prior to punishment, there were significant decreases in activity (negative or “inhibitory” transients) (Fig. 2F [T]; Fig. S2) associated with responses on both the to-be punished and to-be unpunished levers. Our use of the term inhibitory is merely descriptive (see also [26]), and does not imply any specific inhibitory mechanism.

This same inhibitory activity pattern was observed in the first session of punishment (Fig. 2F [P1]). However, when punishment learning was established (Fig. 2F [P5–6]; Fig. S2), BLA transients to punished lever-presses were no longer inhibitory. Instead, there were now significant excitatory transients around punished lever-press. Importantly, this change in BLA transients was response-specific: Ca^2+^ transients to unpunished lever-presses remained unchanged and inhibitory. Within-subject comparisons of punished and unpunished lever-press transients confirmed significant differences between activity around punished and unpunished lever-presses.

Critically, the same pattern of BLA activity around lever-presses was observed during choice test when the punisher was absent (Fig. 2F, S2; *n* = 10). Punished and unpunished lever-press transients were significantly different from each other, with punished and unpunished lever-pressing characterized by significant excitatory and inhibitory transients, respectively. This preservation of excitatory transients during choice test shows that punished lever-press transients were not dependent on recent exposure to aversive outcomes per se or on the trial-based structure of punishment sessions.

#### Midazolam tests

**Fig. 3:**
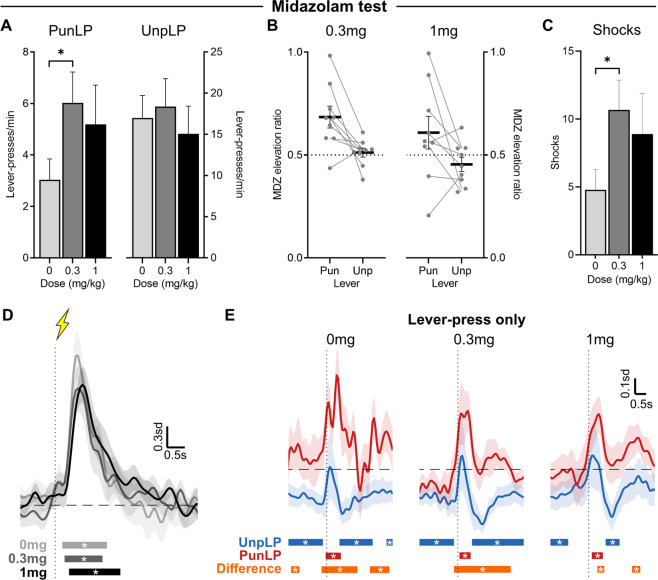
Influence of midazolam (MDZ) on punished behavior and BLA activity. **A** Mean ± SEM punished (left) and unpunished (*right*) lever-press rate across MDZ doses (*n* = 9). **B** Elevation ratio of lever-pressing (relative to 0 mg/kg) for 0.3 mg/kg (left) and 1 mg/kg (right) MDZ; bar = mean ± SEM, dots = individuals. **C** Mean ± SEM shocks received across MDZ doses. **D** Mean ± SEM BLA activity kernel around shock deliveries across MDZ doses. Bars at bottom of graph indicate significant deviations from baseline (dF/F ≠ 0). Vertical dashed line indicates shock onset, horizontal dashed line indicates baseline (dF/F = 0). **E** Mean ± SEM activity kernel around punished (PunLP; red) and unpunished (UnpLP; blue) lever-presses (no outcome) across MDZ tests. Bars at bottom of graph indicate significant deviations from baseline (95% CI) for PunLP and UnpLP, and significant differences between punished and unpunished kernels (orange). Vertical dashed lines indicate time of lever-press, horizontal dashed lines indicate baseline (dF/F = 0).

The identification of punished lever-press transients which emerged across the course of punishment training is consistent with the possibility that BLA activity encodes the learned aversive value of instrumental actions. To test this, we examined the effects of midazolam (MDZ) on these transients and punished behavior. Benzodiazepines have well-documented anti-punishment effects, specifically increasing punished behavior [14]. At higher doses, benzodiazepines can also have sedative effects, reducing behavior generally. Following punishment (Fig. 2), we tested the effect of systemically administered benzodiazepine, midazolam (MDZ), on behavior and BLA activity (Fig. 3). Subjects (*n* = 9) received control (0 mg/kg) and MDZ (0.3 or 1 mg/kg) injections prior to separated punishment sessions (within-subject, order counter-balanced).

Although there was a trend towards increased punished lever-press rates for both doses of MDZ relative to control (Fig. 3A), only 0.3 mg caused a significant and selective increase in punished responding (*F*(1,8) = 19.93, *p* = 0.002); 1 mg did not robustly increase punished responding (*F*(1,8) = 1.12, *p* = 0.320). There was no significant effect of 0.3 mg (*F*(1,8) = 0.727, *p* = 0.419) or 1 mg (*F*(1,8) = 1.738, *p* = 0.224) MDZ on unpunished lever-press rates.

The effect of MDZ on lever-pressing was also assessed via an elevation ratio (Fig. 3B). This normalizes MDZ lever-press rates against control lever-press rates, providing a more sensitive measure of directional, proportional change. When comparing ratios against the null of 0.5 (no difference), 0.3 mg MDZ significantly increased punished lever-pressing (*t*(8)  = 3.485, *p* = 0.008), without commensurately affecting unpunished lever-pressing (*t*(8) = 0.5282, *p* = 0.612). As found using lever-press rates, 1 mg MDZ did not reliably increase punished lever-pressing (*t*(8) = 1.365, *p* = 0.209) or decrease unpunished lever-pressing (*t*(8) = −1.327, *p* = 0.221). This corresponds with previous findings that 0.3 mg/kg MDZ produces selective anti-punishment effects while 1 mg/kg does not due to the dominance of sedative effects [16]. However, anti-punishment effects have been reported for doses above 1 mg/kg [20], suggesting a potential influence of individual differences in sensitivity to MDZ. When considering individual differences in effects of 1 mg MDZ observed here, the two subjects that showed decreased punished lever-pressing at 1 mg MDZ also showed decreased unpunished pressing, indicative of MDZ-induced sedation [19]. These two subjects showed increased punished lever-pressing at 0.3 mg MDZ, but may have been particularly sensitive to MDZ, causing sedative effects to dominate at 1 mg/kg.

These impacts of MDZ on punished behavior were consequential. The increased punished lever-pressing following 0.3 mg MDZ resulted in significantly more shocks delivered (F(1,8) = 19.99, *p* = 0.002) (Fig. 3C).

Interestingly, shock-evoked BLA transients (Fig. 3D) were unaffected by MDZ, suggesting that the anti-punishment effects of MDZ were not due to a change in BLA encoding of the punisher per se. Indeed, across MDZ tests, shocks elicited robust excitatory transients of similar magnitude and duration to those observed in non-MDZ punishment sessions. Punished and unpunished lever-pressing were associated with excitatory and inhibitory BLA transients, respectively (Fig. 3E). However, excitation around the punished lever-press, particularly that immediately preceding the lever-press, was less pronounced under MDZ. Indeed, comparisons of punished lever-press activity across MDZ tests (Figure S3) revealed significantly less activity prior to punished lever-press during 0.3 mg test relative to 0 mg, but no significant differences observed between 1 mg and other doses.

#### Assessing changes in BLA activity across sessions

**Fig. 4:**
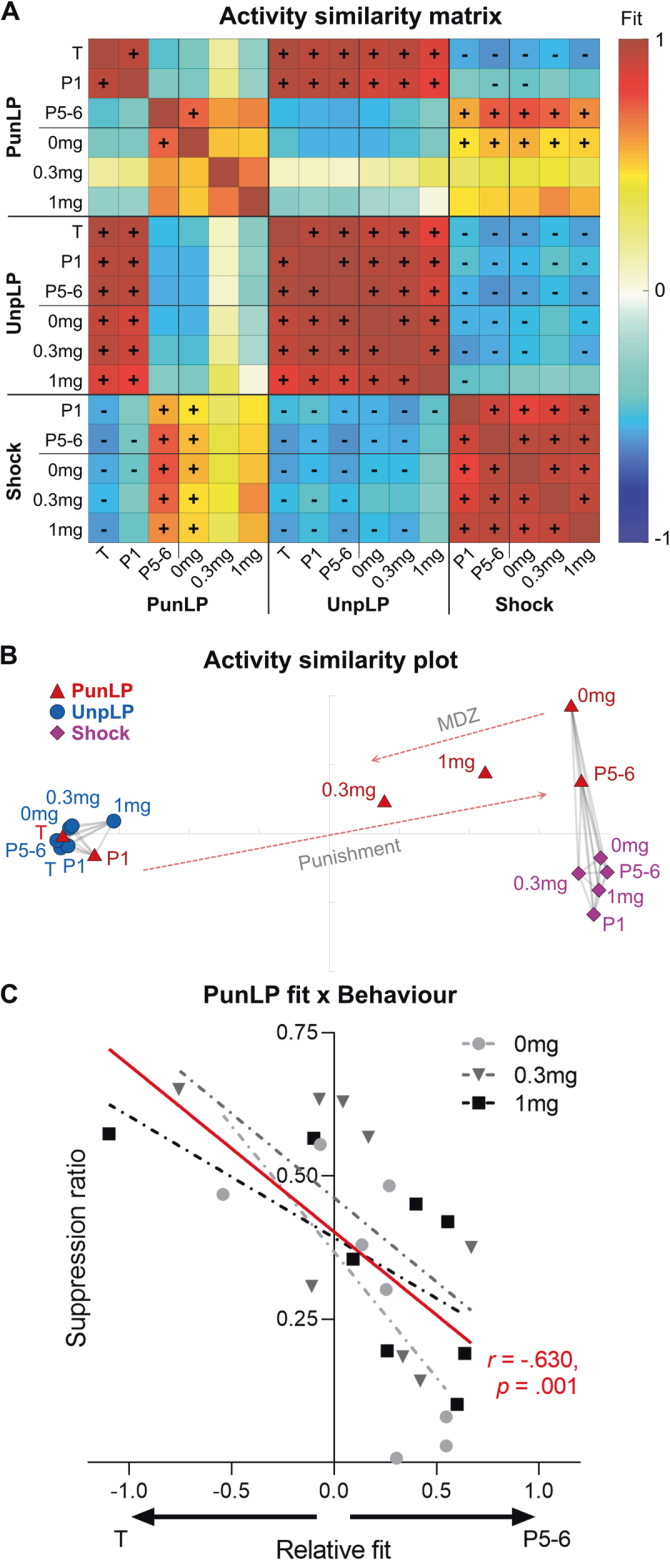
Change in BLA activity across punishment and midazolam tests. **A** Similarity of punished lever-press (PunLP), unpunished lever-press (UnpLP), and shock activity kernels across lever training (T), punishment (P1, P5-6), and MDZ tests (0 mg, 0.3 mg, 1 mg), determined via normalized fit between mean of subject waveforms. Significant positive (+) and negative (−) fits were identified via bootstrapping (95% CI of fits). UnpLP and shock-related activity kernels are relatively unchanged throughout punishment and MDZ tests, whereas PunLP kernels change substantially. **B** 2D plot of kernel similarities (multidimensional scaling of fit distance [1 – fit]). Gray lines connect significantly similar waveforms. Dashed arrows indicate the general effect of punishment and MDZ on PunLP coding in BLA. Punishment transformed PunLP kernels from UnpLP-like kernels into shock-like kernels; MDZ reverted PunLP kernels towards their pre-punishment state. **C** Relationship between subject’s BLA PunLP activity and PunLP behavior across MDZ tests (0 mg, 0.3 mg, 1 mg). Fit of MDZ test PunLP activity onto late punishment vs. pre-punishment PunLP activity was quantified in a relative fit score (P5-6 fit – T fit, per subject). Relative fit was compared against PunLP suppression (suppression ratio relative to T, per subject): less punishment avoidance was associated with PunLP activity kernels being more similar to pre-punishment than late punishment.

To directly compare peri-event BLA activity across punishment learning and MDZ tests, we calculated the fit between punished lever-press, unpunished lever-press, and shock waveforms across sessions. Fit scores quantify the degree to which one waveform is similar to another: positive values indicate matching transients (1 indicates identical waveforms relative to baseline) whereas negative values indicate opposite transients (-1 indicates perfectly inverse waveforms relative to baseline). Significant positive and negative fits were identified through fit confidence intervals bootstrapped from subject mean waveforms. Fits between the various waveforms are depicted in Fig. 4A. The similarity/dissimilarity between waveforms was also depicted in 2D space (Fig. 4B), such that similar waveforms are plotted closer together than dissimilar waveforms. Grey lines connecting datapoints in Fig. 4B indicate significantly positive fits, i.e., activity pattern clusters.

Unpunished lever-presses and shock transient waveforms were stable across punishment and MDZ test sessions. Cross-session fits of unpunished lever-press transients were uniformly high and significantly similar (Fig. 4A; mean fit = 0.938), as were cross-session fits of peri-shock transients (mean fit = 0.914). When comparing fits between unpunished lever-press and shock transients, fits were reliably negative (mean fit = −0.547), reflecting the inhibitory versus excitatory transients observed around these respective events across sessions. Visualizing these relationships in a 2D similarity plot (Fig. 4B) showed that unpunished lever-press transients across sessions occupied a tightly clustered space, while shock transients occupied a separate but similarly tightly clustered space.

In contrast, punished lever-press transients changed substantially across punishment and MDZ sessions. During training and first punishment session, transients around punished lever-presses were highly similar to unpunished lever-pressing and dissimilar to shock transients. However, for later punishment sessions, the reverse became true; punished lever-press transients diverged from unpunished lever-press transients and became significantly similar to shock transients, as highlighted by the rightward arrow in Fig. 4B.

Importantly, MDZ reverted punished lever-press transients towards their pre-punishment state. Punished lever-press transients following control (0 mg) injections were similar to late punishment lever-press and cross-session shock transients. This similarity was undermined by MDZ, with punished lever-press activity following 0.3 mg and 1 mg MDZ no longer showing significant similarity to those transients (Fig. 4A, B). Critically, punished lever-press transients became more similar to pre-punishment/unpunished lever-press transients under MDZ, as highlighted by the leftward arrow in Fig. 4B. This was particularly true for 0.3 mg punished lever-press transients, whose fit against unpunished and pre-punishment lever-press activity trended positive instead of negative (Fig. 4A). It is noteworthy that this stronger effect for 0.3 mg MDZ on BLA punished lever-press activity mirrors the stronger effect of this dose on punished lever-pressing itself.

#### Relationship between anti-punishment effects of MDZ and BLA lever-press transients

These analyses indicate that MDZ increased punished responding and reverted BLA coding of punished behavior towards a pre-punishment state. We investigated the direct relationship between these two effects, particularly given that the anti-punishment effects of MDZ on behavior appeared to be influenced by individual differences.

We first calculated a relative fit score that quantified how strongly each subject’s punished lever-press transients per MDZ test (0 mg, 0.3 mg, 1 mg) fit their punished lever-press activity during late punishment versus pre-punishment. A correspondingly normalized measure of punished behavior was obtained via suppression ratio, which assesses punished response suppression relative to pre-punishment. Analyses of suppression ratios were consistent with previous analyses: punished lever-pressing was suppressed relative to training under saline (mean ± SEM = 0.268 ± 0.072; *t*(8) = 3.243, *p* = 0.012) and 1 mg/kg MDZ (mean ± SEM = 0.343 ± 0.057; *t*(8) = 2.737, *p* = 0.026), but not 0.3 mg/kg MDZ (mean ± SEM = 0.398 ± 0.076; *t*(8) = 1.341, *p* = 0.217), with 0.3 mg/kg MDZ significantly increasing suppression ratio (i.e., punished responding) relative to saline (*t*(8) = 4.301, *p* = 0.003).

Critically, we found a strong negative relationship (*r* = −0.63, *p* = 0.001) between how much punished behavior reverted to pre-punishment levels and how much punished lever-press transients reverted to their pre-punishment form. In other words, the more that punished lever-press transients reverted to their pre-punishment form, the more animals failed to avoid punishment. This inverse relationship was true across MDZ doses, suggesting the anti-punishment effects of MDZ are mediated by, or at least tracked by, the degree to which MDZ influences BLA coding of punished responses.

## Discussion

Punishment involves learning the instrumental contingencies between actions and their adverse consequences. Here we used a well-controlled, within-subjects punishment task to study how BLA supports punishment learning. Rats were trained to respond on two levers for food reward prior to one of those responses being punished with footshock. Punishment was effective. It caused response-specific suppression of lever-pressing, increased the latencies with which rats responded on the punished lever, but did not increase levels of immobility indicative of involuntary conditioned fear responses.

Using fiber photometry, we examined BLA principal neuron Ca^2+^ transients during instrumental punishment and found that BLA activity encodes instrumental aversion. At the population-level, BLA principal neurons exhibited phasic excitatory transients to response-elicited footshock punishers across punishment sessions. Prior to punishment BLA principal neurons exhibited phasic decreases in activity around lever-presses associated with reward. However, across punishment sessions, BLA transients to the punished action became excitatory whereas unpunished actions remained inhibitory. This same profile of within-subject, bidirectional BLA transients was observed during choice tests when the punisher itself was absent. So, there was evidence here for BLA encoding of both instrumental aversive outcomes and specific, punished instrumental actions. This specific BLA encoding of both punished instrumental actions and their consequences supports the view that BLA is a critical neural substrate for instrumental aversion [3, 6, 27].

Our findings suggest that punished responses may evoke punisher-specific representations in BLA to guide behavior. Similarity analyses showed that BLA Ca^2+^ transients to punished instrumental actions became highly similar to the punisher itself across training. This parallels findings from Pavlovian fear conditioning where BLA ensemble activity to Pavlovian CSs becomes more similar to the shock US outcomes they predict [8, 28]. In Pavlovian tasks, increased alignment of BLA activity between antecedents and outcomes has been interpreted in two distinct ways. First, it may reflect common behavioral responses to CSs (i.e., antecedents) and USs (i.e., outcomes) as they come to demand increasingly similar behavioral outputs across conditioning [13]. According to this view, the similarity in BLA Ca^2+^ transients to punished lever-presses and footshock reflect underlying similarity in behavioral responses to them. Although this seems plausible in Pavlovian tasks, it is an unlikely explanation of the present data because lever-presses are not responses to footshock. Second, this increased alignment could reflect BLA encoding of the aversive value common to punished actions and punishers. Indeed, consistent with this, BLA Ca^2+^ transients throughout the task were well-represented in a low dimensional space corresponding to a continuum of aversive valence (Fig. 4). Representations of aversive value are presumed necessary for appropriate punishment avoidance, and interfering with BLA activity, including during the moments of punishment, reduces avoidance [6, 11, 12, 29–32]. Given that punishment learning is putatively underpinned by specific Action–Punisher associations [3, 25], our findings suggest that punished responses may evoke punisher-specific representations in BLA to guide behavior, a function already ascribed to BLA for appetitive outcomes [33].

The results from midazolam tests strengthen this interpretation. Systemic administrations of midazolam not only increased punished responding but also reverted BLA activity around punished responses towards their unpunished state. Critically, BLA activity around footshock and unpunished responses were relatively unaffected by midazolam. So, the selective effects of midazolam on punishment were linked to selective effects on BLA coding of punished actions. This shows that benzodiazepines do not undermine aversion coding in BLA generally [34, 35], but rather they interfere with the response-specific representations promoting punishment. Critically, individual differences in the effects of midazolam on punishment avoidance were directly predicted by the degree to which the benzodiazepine could revert lever-press activity to its pre-punishment state. This suggests that individual differences in the anxiolytic action of benzodiazepines, traditionally assessed using punishment tasks [14], may be linked to individual variation in this reversion.

There are three methodological limitations worth considering. First, the population read-out obtained here using fiber photometry prevents inferences about the activity of individual BLA neurons. BLA neurons can exhibit marked activity heterogeneity to stimuli and behaviors [13, 36]. Moreover, both the activity and functions of BLA principal neurons can be dissociated according to their specific projection targets [37]. So, whether and how the activity we observed to outcomes and punished versus unpunished responses were driven by overlapping or separate neuronal ensembles remains unclear. How punishment is encoded across BLA ensembles, and how these in turn influence broader network activity to guide behavior, is an important area for future research. Second, we administered midazolam systemically, so the effects observed here may be mediated by actions in brain regions beyond BLA. It is worth emphasizing that the effect of benzodiazepines on punishment avoidance are linked to, and directly recapitulated by, its actions within BLA [15, 38]. However, other sites of action such as hippocampus may contribute to the effects of benzodiazepines on behavior and BLA activity [35, 39, 40]. Third, we studied only male rats here, so the nature, role, and relevance of sex differences in punishment remain worth investigating. It is worth noting sex differences using immediate punishment tend to be modest and attributable to increased propensity to fear over punishment learning in females [4, 17, 41, 42]. Moreover, our rodent work, based largely on male rats, accurately accounts for punishment learning in female and male humans [43].

In summary, we investigated BLA principal neuron activity across punishment learning, choice, and under the influence of benzodiazepine. We show that instrumental punishment is encoded in BLA activity via excitations to punishers and their behavioral antecedents, in contrast to negative deviations around unpunished actions. Benzodiazepine increased punished behavior, but only to the extent it reverted BLA coding of punished actions to their pre-punishment state. Together, these findings show that punishment learning is supported by aversion-coding of instrumental responses in the BLA and that the anti-punishment effects of benzodiazepines are associated with a reversion of this aversion coding.

## Supplementary information

Supplemental Material
