# Supplementary material for: Instrumental aversion coding in the basolateral amygdala and its reversion by a benzodiazepine
Source: Neuropsychopharmacology. 2021 Sep 7;47(6):1199–209. doi: 10.1038/s41386-021-01176-2 (PMC9018846; doi:10.1038/s41386-021-01176-2)
Supplement: Supplementary file 1 — Supplemental Material [file 41386_2021_1176_MOESM1_ESM.pdf]

SUPPLEMENTAL MATERIAL

Materials and Methods

Apparatus

The floors consisted of steel rods (4mm diameter, 15mm apart) connected to a constant-current generator. The center of the right side-wall included a recess (5 x 3 x 15 cm) that housed a magazine dish (3 cm diameter) into which 45mg grain pellets (Bio-Serv, NJ, USA) were delivered. The magazine was flanked by retractable levers (2 x 4 cm). All events were controlled via Med-PC IV software (Med Associates, St Albans, VT, USA). A digital camera was installed to record animals' spatial positions. For fiber photometry, a patch cable threaded into the chamber through a ceiling hole and supported by a counter-weighted gimbal holder. Multimode fiber optic cannulae implants and patch cables (0.39 NA, 400µm core) were constructed using materials from Thor Labs (Newton, NJ, USA).

Two Doric LEDs, controlled via dual channel LED drivers, provided 465 nm (Ca^{2+}-dependent signal) and 405 nm (isosbestic control signal) excitation light. GCaMP fluorescence wavelengths (~525 nm, ~ 430 nm) were measured using femtowatt photoreceivers (Newport 2151). Doric Dual Fluorescence Mini Cube (FMC2, Doric Lenses) relayed excitation/fluorescence wavelengths to/from pre-bleached patch cable and fiber optic implant. A real-time processor (RZ5P, TDT) controlled and modulated excitation lights (465 nm: 209 Hz; 405 nm: 331 Hz), as well as demodulated and low-pass filtered (3 Hz) fluorescence signals. The RZ5P also received Med-PC signals to record behavioral events in real-time. Light intensity at the tip of the patch cable was maintained at 10-40 µW across sessions.

Surgery

Rats were anaesthetized with 1.3 ml/kg ketamine (100 mg/ml; Ketapex; Apex Laboratories, Sydney, Australia) and 0.2 ml/kg muscle relaxant, xylazine (20 mg/ml; Rompun; Bayer, Sydney, Australia) (i.p.) and placed in stereotaxic apparatus (Model 942, Kopf, Tujunga, CA), with the incisor bar maintained at approximately 3.3 mm below horizontal to achieve a flat skull position. A 5µl, 30-gauge conical tipped microinfusion syringe (Hamilton; Reno, NV, USA) was used to inject AAV vector (0.75µl; 0.25µl/min) into BLA (AP −2.9, ML ±5.0, DV −8.2 mm from bregma; [19]). The syringe remained at the injection site for 5min to allow diffusion. A 400µm optic fiber was implanted above BLA (AP −2.9, ML ±5.0, DV −8.0 mm from bregma) and anchored in position with dental cement and jeweller's screws. Immediately following surgery, animals were given i.p. injections of antibiotics (0.3ml procaine penicillin solution [300mg/ml Benicillin; Illium], 0.3ml cefazolin [100mg/ml]).

Immunohistochemistry

Rats were anesthetized with i.p. injections of sodium pentobarbital (100mg/kg) and perfused with 0.9% saline solution containing 1% sodium nitrate and heparin (5000 IU/ml), followed by phosphate buffer solution (PB; 0.1M) with 4% paraformaldehyde. Brains were extracted, incubated in 20% sucrose solution for cryoprotection, sliced coronally (40µm) using a cryostat and stored in PB solution with 0.1% sodium azide at 4°C.

Fiber placement and GCaMP expression were determined using fluorescent immunohistochemistry. Brain tissue was washed in PB, incubated in PBT-X solution (10% horse serum [NHS], 0.5% Triton X-100 in PB) for 2 hours, and then incubated in PBT-X solution (2% NHS, 0.2% TritonX-100 in PB) with primary antibody (1:1000 polyclonal rabbit anti-GFP, ThermoFisher Scientific, #A11122) at room temperature for 24hr. Tissue was washed with PB and incubated overnight in PBT-X (2% NHS, 0.2% TritonX-100 in PB) with secondary antibody (1:1000 AlexaFluor 488-conjugate anti-rabbit, ThermoFisher Scientific). Tissue was washed with PB and mounted onto gelatinised slides. Slides were left to dry and then cover-slipped. GCaMP6f expression and cannula placements were verified using fluorescent microscopy. Animals were excluded from analyses if fiber tip and GCaMP expression could not be confirmed as co-localized in BLA; n = 14 animals were included across analyses.

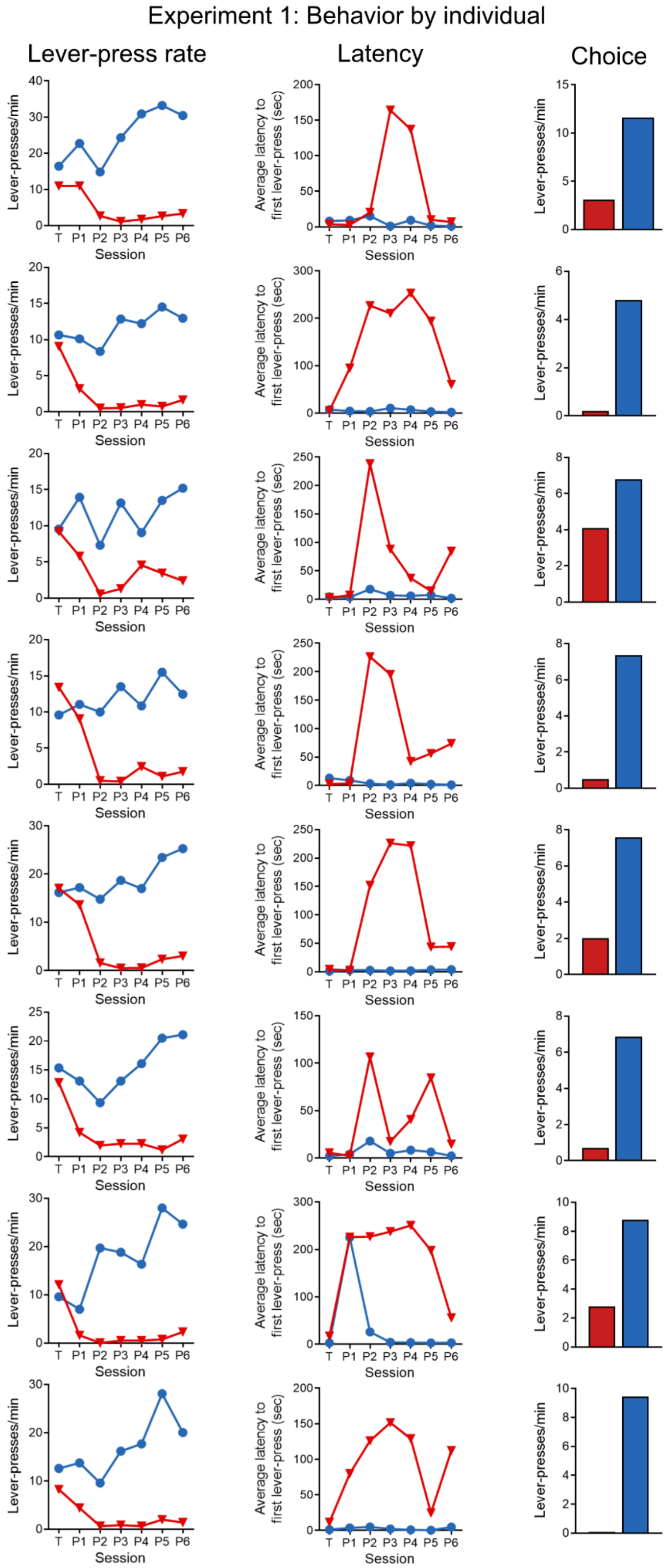

Figure S1. Behavior across individuals in Experiment 1. Each row represents data from an individual rat. *Left column*: lever-press rate across punishment. *Middle column*: average latency to first lever-press across trials. *Right column*: lever-press rate during choice test.

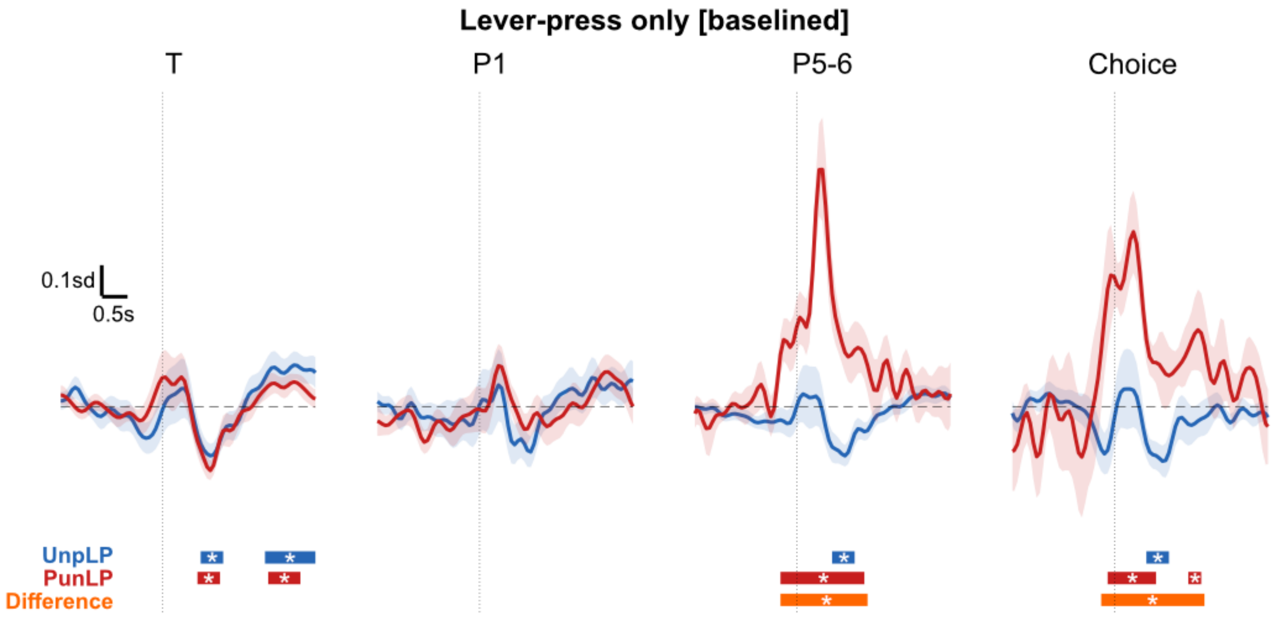

Figure S2. Baselined analysis of lever-press activity in basolateral amygdala. Mean±SEM BLA activity kernel (baselined to -5 to -3 sec per trial) around punished (PunLP; red) and unpunished (UnpLP; blue) lever-presses (no outcome) across training (T), punishment (P1, P5-6) and choice test. Bars at bottom of graph indicate significant deviations from baseline (95% CI) for PunLP and UnpLP, and significant differences between punished and unpunished kernels (Difference; orange). Vertical dashed lines indicate time of lever-press, horizontal dashed lines indicate baseline (dF/F = 0).

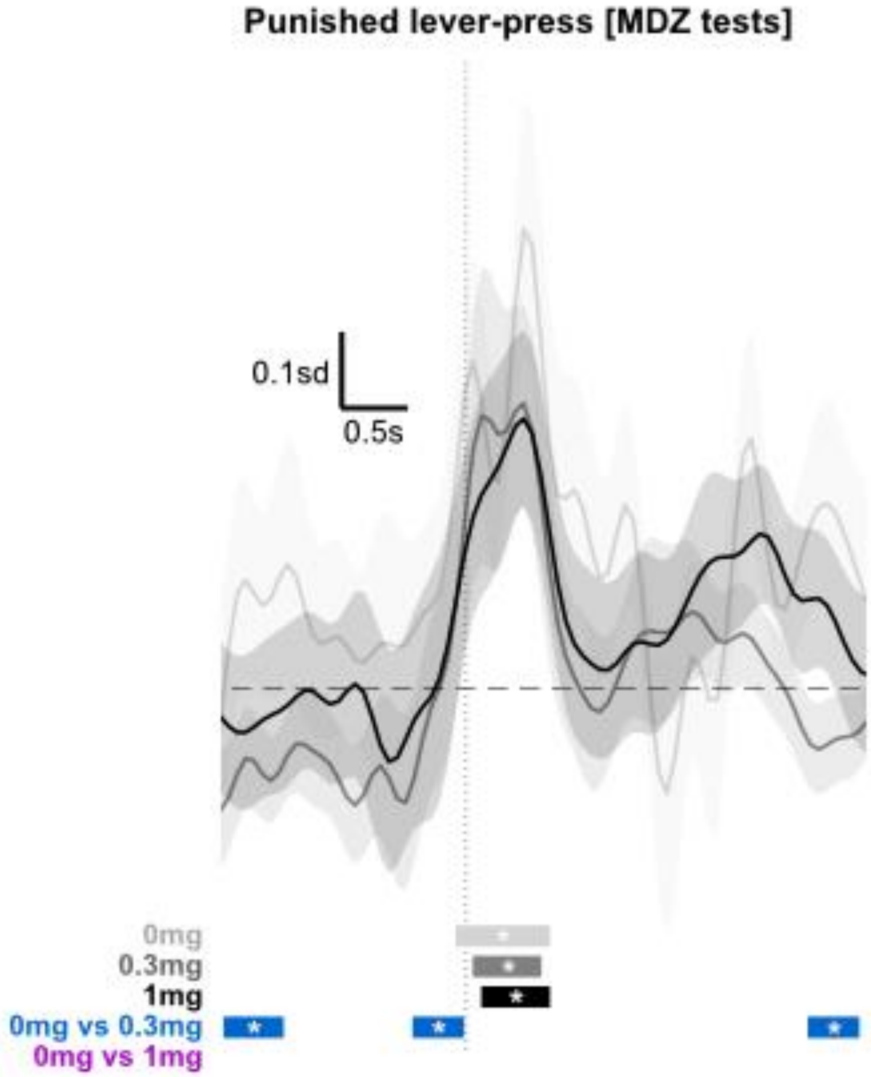

Figure S3. Comparison of punished lever-press activity across midazolam tests. Mean±SEM activity kernel around punished lever-presses (no outcome) across midazolam (MDZ) tests. Vertical dashed lines indicate time of lever-press, horizontal dashed lines indicate baseline (dF/F = 0). Bars at bottom of graph indicate significant deviations from baseline (95% CI) during 0mg, 0.3mg and 1mg tests, and significant differences in activity between tests (within-subject comparison). Only 0mg versus 0.3mg tests showed significantly different punished lever-press activity.
